# 104. Accuracy of ChatGPT for UTI Diagnosis and Management Questions

**DOI:** 10.1093/ofid/ofad500.020

**Published:** 2023-11-27

**Authors:** Kalpana Gupta, William O’Brien, Judith Strymish

**Affiliations:** VA Boston HCS and Boston University School of Medicine, West Roxbury, MA; VA Boston Healthcare System, West Roxbury, MA; VA Boston Healthcare System, West Roxbury, MA

## Abstract

**Background:**

Artificial Intelligence chatbots such as ChatGPT are increasingly being utilized for medical information by the public as well as by medical professionals, including researchers. We investigated the accuracy of information and citations generated by this search engine for a common medical condition, UTI.

**Methods:**

This was a qualitative pilot evaluation of an artificial intelligence chatbot for answering and referencing clinical questions on UTI. We created 5 clinical questions (Table) related to UTI diagnosis and treatment. Two clinical infectious diseases experts graded the responses on a Likert scale as to accuracy of the response. The gold standard for responses was derived from IDSA guidelines and/or evidence based electronic resources (e.g. UpToDate). Citations were checked for accuracy using PubMed searches, Google Scholar and also, in one case, direct communication with the purported author.

ChatGPT Performance on UTI Questions

**Figure:**
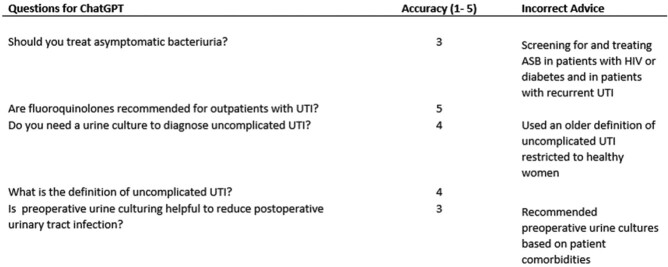

**Results:**

ChatGPT answered 3 of the 5 questions mostly correctly (Likert scale 4 out of 5). The response to one question was rated as only half correct (Likert scale 3 out of 5). One question was answered 100% correctly. Incorrect information was primarily related to diagnostic approaches to urine cultures for both asymptomatic and symptomatic patients (Table), and did not reflect answers found in current guidelines.

When prompted for supportive evidence of answers, ChatGPT created plausible, yet fabricated studies. The authors were real and well-known experts in the field, but the combinations of authors, journal title and paper title were often false. Citations were incorrect for all 5 questions and fabricated in 12 of 24 provided citations.

**Conclusion:**

ChatGPT performed surprisingly well on correctly answering basic diagnosis and management questions on UTI, a commonly encountered condition with a large body of literature to train from. The lack of current information from guidelines hampered accuracy and could be a barrier to appropriate use of this technology for real-time learning or clinical decision-making. The inaccuracy of citations limits its use for supporting evidence-based research and publications.

**Disclosures:**

**Kalpana Gupta, MD MPH**, GSK: Advisor/Consultant|Iterum Therapeutics Limited: Advisor/Consultant|Qiagen, Inc.: Advisor/Consultant|UpToDate Wolters Kluwer Health: Royalties

